# Large Language Model and Knowledge Graph-Driven AJCC Staging of Prostate Cancer Using Pathology Reports

**DOI:** 10.3390/diagnostics15192474

**Published:** 2025-09-27

**Authors:** Eunbeen Jo, Tae Il Noh, Hyung Joon Joo

**Affiliations:** 1 Department of Biomedical Informatics, Korea University College of Medicine, Seoul 02841, Republic of Korea; goodsoy@korea.ac.kr; 2 Department of Urology, Korea University School of Medicine, Seoul 02841, Republic of Korea; 3 Korea University Research Institute for Medical Bigdata Science, Korea University, Seoul 02841, Republic of Korea; 4 Department of Cardiology, Cardiovascular Center, Korea University College of Medicine, Seoul 02841, Republic of Korea

**Keywords:** artificial intelligence, natural language processing, prostate cancer, pathology

## Abstract

**Background/Objectives**: To develop an automated American Joint Committee on Cancer (AJCC) staging system for radical prostatectomy pathology reports using large language model-based information extraction and knowledge graph validation. **Methods**: Pathology reports from 152 radical prostatectomy patients were used. Five additional parameters (Prostate-specific antigen (PSA) level, metastasis stage (M-stage), extraprostatic extension, seminal vesicle invasion, and perineural invasion) were extracted using GPT-4.1 with zero-shot prompting. A knowledge graph was constructed to model pathological relationships and implement rule-based AJCC staging with consistency validation. Information extraction performance was evaluated using a local open-source large language model (LLM) (Mistral-Small-3.2-24B-Instruct) across 16 parameters. The LLM-extracted information was integrated into the knowledge graph for automated AJCC staging classification and data consistency validation. The developed system was further validated using pathology reports from 88 radical prostatectomy patients in The Cancer Genome Atlas (TCGA) dataset. **Results**: Information extraction achieved an accuracy of 0.973 and an F1-score of 0.986 on the internal dataset, and 0.938 and 0.968, respectively, on external validation. AJCC staging classification showed macro-averaged F1-scores of 0.930 and 0.833 for the internal and external datasets, respectively. Knowledge graph-based validation detected data inconsistencies in 5 of 150 cases (3.3%). **Conclusions**: This study demonstrates the feasibility of automated AJCC staging through the integration of large language model information extraction and knowledge graph-based validation. The resulting system enables privacy-protected clinical decision support for cancer staging applications with extensibility to broader oncologic domains.

## 1. Introduction

The American Joint Committee on Cancer (AJCC) staging manual is the benchmark for determining cancer classification, prognosis, and treatment approaches [1]. The 8th edition of the AJCC manual contains all available information on adult cancer staging [1,2]. The staging of prostate cancer is based on the integration of the Tumor-Node-Metastasis (TNM) staging system, grade group derived from Gleason scores (GS) according to the International Society of Urological Pathology (ISUP) 2014 consensus guidelines, and prostate-specific antigen (PSA) level [2,3].

Radical prostatectomy (RP) has been established as an effective therapy for patients with moderate-risk and high-risk prostate cancer [4,5]. In patients treated with RP as primary therapy, the pathologic features of the prostatectomy specimen are major determinants of prognostic assessment and of the appropriateness of adjuvant therapy [6]. Appropriate pathological examination of surgical specimens by pathologists is critical for accurate prognostic assessment and optimal patient management [7]. For RP specimens, the International Collaboration on Cancer Reporting (ICCR) recommends structured pathology reporting using standardized ICCR datasets with required (core) and recommended (non-core) elements [8]. Core macroscopic elements include specimen weight, seminal vesicles, and lymph nodes, whereas core microscopic elements include histologic tumor type and grade, extraprostatic extension (EPE), and seminal vesicle invasion (SVI) [8,9]. Clinical information and PSA levels are categorized as recommended data elements [8,9].

Most pathology reports are written in free-text format [9,10], which poses significant challenges for information extraction and clinical decision support. Large language models (LLMs) have demonstrated significant advances in medical applications [11,12], including information extraction from electronic health records [13], clinical text summarization [14], and document classification [15]. Prompt engineering utilizing LLMs enables the extraction of structured information from narrative pathology reports without requiring manual annotation or model training [10,16]. Medical knowledge graphs systematically represent information by organizing medical concepts and events as nodes and edges to describe their interrelationships [17]. This study aims to develop an automated AJCC staging system for RP pathology reports by applying prompt engineering with LLMs for information extraction and knowledge graph-based approaches for validation and staging classification. Specifically, key pathological information is extracted from free-text reports, a knowledge graph is constructed from the extracted data, and rule-based AJCC staging is implemented with integrated data consistency verification.

## 2. Materials and Methods

### 2.1. Dataset and Expansion of Data Elements

**Figure 1:**
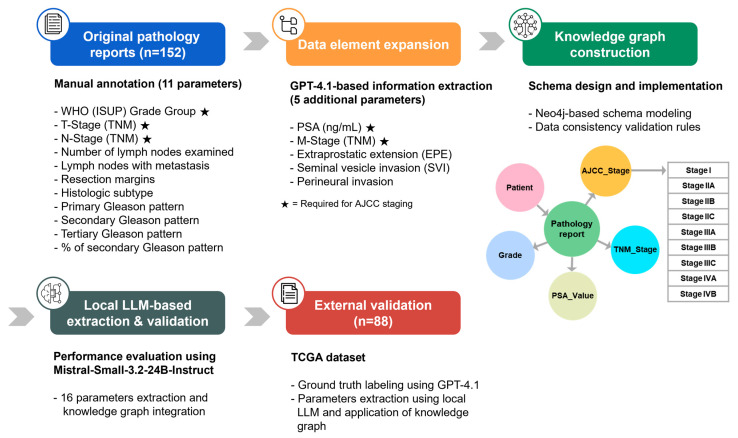
Overview of the automated AJCC staging system workflow. The integrated system employs a multi-step pipeline involving LLM-based information extraction, missing data imputation, knowledge graph construction, and validation.

Figure 1 illustrates the overall study workflow. For the purpose of extracting structured data, performing AJCC staging, and validating data consistency, an anonymized, retrospective dataset consisting of 579 English pathology reports from 340 prostate cancer patients was used [10]. The final dataset consisted of 152 cases in which PSA values were documented in the pathology reports. The dataset was publicly released on Zenodo for academic research purposes [18]. For patients with multiple pathology reports, the most recent report was used as the reference. Missing variables were supplemented using earlier reports, and once a value was identified, it was adopted as the final value.

The pathology reports were manually annotated with 11 parameters selected based on their clinical and prognostic relevance [10]. In this study, additional extraction was performed using GPT-4.1 to extract five specific data elements not included in the original annotations: PSA level, M-stage, EPE, SVI, and perineural invasion. Information extraction was performed using the GPT-4.1 model accessed through the OpenAI API (OpenAI, L.L.C., San Francisco, CA, USA, 17 July 2025, https://openai.com/) with a zero-shot prompting approach. The model output was provided in a structured JSON format containing the extracted information, the supporting evidence text span, and a confidence score ranging from 0 to 1. All extracted data were manually reviewed to confirm accuracy, and the prompt used for extraction is provided in Appendix A.1.

To evaluate the generalizability of the proposed approach, the publicly available The Cancer Genome Atlas (TCGA)-Reports dataset from Mendeley Data was used as an external validation dataset [19,20]. From 9523 unstructured free-text pathology reports representing 32 tissue or cancer types from a wide variety of institutions, 300 reports containing ‘radical prostatectomy’ were identified. Among these, 88 cases with documented PSA values were selected as the final external validation dataset. All 16 parameters for the external dataset were annotated using GPT-4.1 (accessed 14 September 2025) with a zero-shot prompting approach. The extracted data were manually reviewed, and the prompt used for annotating the external dataset is provided in Appendix A.2.

### 2.2. AJCC Stage Labeling

M-stage was recorded in only 3 cases (2.0%) in the internal dataset and 27 cases (30.7%) in the external dataset. To determine the stage of metastasis, additional clinical data such as the results of systemic imaging studies (bone scan, CT, or PET/CT) are required [21,22]. Because RP is performed for localized prostate cancer, cases with missing M-stage information were classified as M0 stage [23,24,25]. A rule-based AJCC staging algorithm consistent with the 8th-edition criteria [26] was then applied to assign final stage labels to both the internal and external validation datasets. All assigned labels were manually reviewed.

The overall distribution of AJCC stages between datasets was compared using Fisher’s exact test. For stage-specific comparisons of proportions, either Fisher’s exact test or Pearson’s chi-squared test was applied. For each stage within each dataset, 95% confidence intervals for proportions were calculated using the Wilson method. Statistical analyses were performed using R version 4.2.3 (R Foundation for Statistical Computing, Vienna, Austria).

### 2.3. Knowledge Graph Construction

Neo4j (Neo4j, Inc., San Mateo, CA, USA, v 5.28.1) was employed to systematically model the relationships between the extracted pathological variables and their AJCC classifications, yielding a knowledge graph that supports rule-based staging classification and automated data consistency validation. The constructed knowledge graph processes pathology report data through a structured pipeline and exports both classification results and consistency errors to a single Excel file.

#### 2.3.1. Schema Modelling and Node Structure

**Figure 2:**
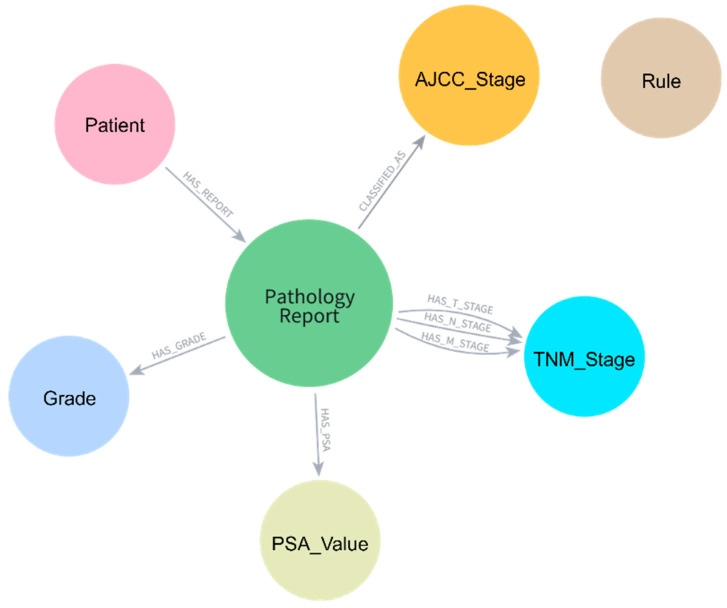
Knowledge graph schema for pathology report data representation. Central Pathology_Report nodes are connected to Patient, AJCC_Stage, TNM_Stage, Grade, and PSA_Value nodes through specific relationship types, enabling structured data storage and rule-based AJCC staging.

The knowledge graph schema was designed to represent the complex relationships between pathological entities and their staging implications (Figure 2). Five primary node types were implemented: Patient, Pathology Report, TNM_Stage, Grade, PSA_Value, and AJCC_Stage. Patient nodes served as the root entities, connected to Pathology Report nodes through HAS_REPORT relationships. Each Pathology Report node stored comprehensive pathological features, including histologic subtype, resection margins, lymph node examination results, Gleason patterns, and invasion indicators (EPE, SVI, and perineural invasion).

TNM staging information was modeled as separate nodes with unique constraints based on both code and type combinations to ensure proper node uniqueness. The schema employed specific relationship types (HAS_T_STAGE, HAS_N_STAGE, HAS_M_STAGE) to connect Pathology Report nodes to their respective TNM_Stage nodes. Grade Group information was represented through dedicated Grade nodes linked through HAS_GRADE relationships, while PSA values were stored in PSA_Value nodes. The final AJCC stage classification was connected through CLASSIFIED_AS relationships to AJCC_Stage nodes.

#### 2.3.2. AJCC Staging Classification

To construct a knowledge graph capable of classifying AJCC stages, a hierarchical rule-based algorithm was implemented following the AJCC 8th edition criteria [26], with conditions applied in the sequential order specified in Table 1. When M-stage information was missing, M0 was assumed for staging classification. Cases that could not be classified due to missing other essential elements for AJCC staging were labeled as “Unknown” with the missing elements specified in parentheses (e.g., Unknown (Missing N-Stage, Grade Group)).

#### 2.3.3. Consistency Validation

The knowledge graph included consistency validation rules to identify potential data extraction errors or clinical inconsistencies. The validation rules examined multiple pathological relationships: T-stage consistency with EPE and SVI, N-stage alignment with lymph node metastasis counts, and Gleason score concordance with the World Health Organization (WHO) Grade Groups according to ISUP 2014 guidelines [27].

Specific validation rules detected the following inconsistencies: (1) T2 stages with EPE present; (2) presence of SVI in T2 or T3a stages; (3) N0 stages with positive lymph nodes or N1 stages without positive nodes; (4) mismatches between Gleason sum and Grade Group assignments; (5) T3b stages without SVI or T3a stages without EPE [6]. When inconsistencies were identified, the validation rules generated descriptive error codes (e.g., ‘T2_stage_with_EPE_should_be_T3a’, ‘N0_stage_but_positive_lymph_nodes’) and compiled these into dedicated Excel sheets, facilitating systematic data review and accuracy assessment.

### 2.4. LLM-Based Information Extraction and Knowledge Graph Application

#### 2.4.1. Information Extraction Using Open-Source LLM

In this study, although anonymized data was used, considering the privacy protection aspects of medical data, locally available LLM was used to extract information on 16 parameters from pathology reports included in the internal and external validation datasets and evaluate performance. Considering available resources, the open-source LLM was selected as the Mistral-Small-3.2-24B-Instruct (https://huggingface.co/mistralai/Mistral-Small-3.2-24B-Instruct-2506 (accessed on 17 July 2025)) [28] model, which provides performance comparable to Llama 3.3 70B while utilizing significantly fewer computational resources for extracting cancer-related medical attributes from pathology reports [29]. The model was implemented on dual RTX 3090 GPUs. A zero-shot prompting approach was used to extract 16 parameters from the reports and to return the results in a structured JSON format; the prompt template is provided in Appendix A.3. Extraction performance was assessed against manually curated labels with accuracy, precision, recall, and F1-score: accuracy represents the proportion of correct predictions among all predictions; precision represents the proportion of correctly extracted values among all extracted values; recall represents the proportion of correctly extracted values among all ground truth values; and F1-score is the harmonic mean of precision and recall.

#### 2.4.2. Knowledge Graph–Based AJCC Staging and Consistency Validation

The constructed knowledge graph was utilized to perform automated AJCC staging classification and consistency validation using the data extracted by the Mistral-Small-3.2-24B-Instruct model. LLM-extracted information was applied to the knowledge graph to evaluate the clinical applicability of this automated pipeline. In the internal dataset, two reports failed JSON conversion due to excessive text length and were excluded from knowledge graph processing. The LLM-extracted data were integrated into the knowledge graph system, and both staging classification and data consistency validation were performed. For evaluation purposes, cases labeled as “Unknown” with specific missing parameters in parentheses were reclassified as a unified “Unknown” category. Staging classification performance was evaluated against ground truth annotations using accuracy, precision, recall, and F1-score, while consistency validation rules were applied to detect data extraction errors.

## 3. Results

### 3.1. Dataset Characteristics

Table 2 presents the distribution of AJCC staging parameters across the internal (*n* = 152) and external validation (*n* = 88) datasets. The WHO Grade Group distribution differed significantly between datasets (*p* < 0.001), with the internal dataset showing a higher proportion of Grade Group 2 (46.1% vs. 34.1%) and Grade Group 3 (31.6% vs. 25.0%), while the external dataset had higher proportions of Grade Groups 4 (14.8% vs. 2.6%) and 5 (19.3% vs. 13.2%). T-stage distribution also varied significantly (*p* = 0.014). The internal dataset contained more pT2c cases (47.4% vs. 33.0%), whereas the external dataset showed higher rates of pT3a (38.6% vs. 30.9%) and pT3b (23.9% vs. 14.5%). Notably, three pT4 cases (3.4%) were present only in the external dataset.

N-stage distribution showed no significant difference (*p* = 0.090), with the majority being pN0 in both datasets (73.7% internal, 81.8% external). Mean serum PSA levels were significantly higher in the internal dataset (11.9 ng/mL, 95% CI: 8.0–17.2) compared to the external dataset (7.11 ng/mL, 95% CI: 5.0–10.1, *p* < 0.001). M-stage information was rarely recorded in both datasets, with only 3 cases (2.0%) identified in the internal dataset and 27 cases (30.7%) in the external dataset.

Among pathological invasion parameters, EPE was present in 44.1% of internal dataset cases versus 65.9% of external dataset cases (*p* = 0.002). SVI was documented as present in 15.1% and 26.1% of cases in the internal and external datasets, respectively (*p* = 0.055). Perineural invasion showed similarly high rates of presence in both datasets (91.4% internal vs. 90.9% external, *p* = 0.948). Representative examples of GPT-4.1 extraction output with confidence scores and evidence spans for data element expansion are provided in Table A1 (Appendix A). Examples of output for annotating external validation datasets are presented in Table A2 (Appendix A).

Table 3 shows the distribution of AJCC stages between the internal and external validation datasets. The overall stage distribution differed significantly between datasets (*p* = 0.048). The internal dataset showed no Stage IIA cases, while the external dataset contained 3 cases (4.2%). Stage IIB was more common in the internal dataset (28.7% vs. 20.8%), whereas Stage IIIB showed higher prevalence in the external dataset (43.1% vs. 31.1%). Stage IIIA was present only in the internal dataset (5.7%, *p* = 0.048). The remaining stages (IIC, IIIC, and IVA) showed similar distributions between datasets.

### 3.2. Information Extraction Performance

The Mistral-Small-3.2-24B-Instruct model was applied to 150 pathology reports to extract 16 clinical parameters in the internal dataset, resulting in 2400 field-document pairs. Overall micro-averaged performance showed accuracy of 0.973, precision of 0.992, recall of 0.981, and F1-score of 0.986 (Table 4).

M-stage and SVI achieved F1-scores of 1.000, followed by Lymph Nodes with Metastasis (0.997), Tertiary Gleason Pattern (0.997), and N-stage (0.997). Staging-related parameters demonstrated F1-scores of 1.000 (M-stage), 0.997 (N-stage), and 0.980 (T-stage). Percentage of Secondary Gleason Pattern showed the lowest performance, with accuracy of 0.907 and F1-score of 0.951. For example, given “Gleason score 4 + 3 = 7b (60% pattern 4),” the model correctly extracted 60% but failed to compute the secondary proportion of 40%.

The same model was applied to 88 pathology reports to extract 16 clinical parameters from the external validation dataset, resulting in 1408 field-document pairs. Overall micro-averaged performance showed accuracy of 0.938, precision of 0.990, recall of 0.947, and F1-score of 0.968 (Table 5).

Multiple parameters achieved F1-scores of 1.000: Lymph Nodes with Metastasis, N-stage, Number of Lymph Nodes examined, Perineural Invasion, SVI, and T-stage. M-stage showed an F1-score of 0.819, while Percentage of Secondary Gleason Pattern had the lowest performance at 0.803.

### 3.3. AJCC Staging Classification Performance

AJCC staging classification performance in the internal dataset showed macro-averaged precision of 0.922, recall of 0.944, and F1-score of 0.930 (Table 6). Stage IIIC achieved the highest F1-score of 1.000, followed by IVA (0.960), IIB (0.957), and IIIB (0.950). Stage IIC showed lower performance with an F1-score of 0.897, while “Unknown” cases demonstrated the lowest performance with an F1-score of 0.815.

In the external validation dataset, macro-averaged performance metrics for AJCC staging classification were precision 0.830, recall 0.883, and F1-score 0.833 (Table 7). Stage IIIB demonstrated the highest F1-score of 0.939, followed by IIC (0.923) and IVA (0.900). Stage IIA achieved an F1-score of 0.857, while IIB and IIIC showed F1-scores of 0.867 and 0.889, respectively. “Unknown” cases exhibited notably reduced performance with an F1-score of 0.455, representing the lowest performance across all categories. This was mainly attributable to discrepancies in M-stage labeling, where the ground truth was recorded as “pMx” but the open-source LLM extracted the value as “not mentioned,” resulting in misclassification as “Unknown.”

### 3.4. Consistency Validation of Extracted Data

Knowledge graph-based validation applied to LLM-extracted data from 150 internal dataset cases identified 5 cases (3.3%) with staging and grade inconsistencies (Table 8). The most frequent inconsistency was the T3a stage documented without evidence of EPE (2 cases, 40.0%). Other inconsistencies included Gleason 7 inconsistent with Grade Group 3 (1 case, 20.0%), presence of SVI that should correspond to T3b or higher stage (1 case, 20.0%), and Gleason 7 inconsistent with Grade Group 2 (1 case, 20.0%). No staging or grade inconsistencies were detected in the external validation dataset.

The T3a_stage_but_no_EPE cases involved complex pathological descriptions where frozen section findings documented “small capsular defect” with “tumor infiltrates forming margins,” indicating EPE that is classified as pT3a according to the AJCC system [30]. However, the LLM did not reflect these microscopic findings and extracted EPE as “Absent.” The Gleason_7_inconsistent_with_GG3 case occurred when the LLM correctly extracted Primary and Secondary Gleason patterns (3 and 4) but incorrectly identified Grade Group 3 instead of Grade Group 2 from a tabulated format where the correct grade was marked with an “X” symbol, demonstrating challenges in interpreting structured table formats within narrative reports.

## 4. Discussion

This study presents an integrated automated AJCC staging system that combines LLM-based information extraction, missing data imputation, and knowledge graph construction for radical prostatectomy pathology reports. The approach utilized prompt engineering with an open-source LLM to extract structured data, achieving a micro-averaged F1-score of 0.986 in the internal dataset and 0.968 in the external dataset. Grothey et al. [10] demonstrated that open-source LLMs achieve comparable accuracy to third-party managed models such as GPT-4 in extracting structured information from pathology reports. These results suggest that open-source LLMs can effectively extract information from unstructured pathology reports while mitigating privacy concerns. Knowledge graph-based validation identified data errors in 5 of 150 internal datasets (3.3%). These errors were found in cases with complex microscopic descriptions or tabulated formats. For the AJCC staging classification, the system achieved a macro-averaged F1-score of 0.930 in the internal dataset and 0.833 in the external dataset. This pipeline provides a framework for automated clinical decision support systems in cancer staging workflows, processing unstructured free-text medical data into structured formats by validating data accuracy within pathology reports and performing AJCC classification, thereby contributing to systematic clinical data management.

Richter et al. [9] analyzed pathology reports for RP specimens, comparing narrative reports with standardized synoptic reports, and found that PSA values were documented in fewer than half of cases in both formats. In this study, PSA values were available in only 152 of 340 internal pathology reports (44.7%) and 88 of 300 external reports (29.3%), and only these subsets were included in the study. The presence of PSA within pathology reports is not determined by the reporting format [9]. Clinical parameters such as PSA depend primarily on whether the ordering clinician supplies them on the histopathology requisition [9,31]. Therefore, AJCC staging based on pathology reports could be further improved by enhancing documentation practices and information transfer within clinician–pathologist communication workflows, or by integrating pre-diagnostic PSA data from patients into the system.

This study has several limitations. First, although both internal and external validation were performed, the system has not yet been integrated into real clinical workflows. In particular, only pathology reports were available, and relevant clinical data such as imaging findings could not be incorporated. Cases without documented M-stage in pathology reports were classified as M0, given that radical prostatectomy is performed for localized prostate cancer [23,24,25]. Future research should integrate clinical data, such as systemic imaging results or PSA levels, into the pipeline for application in clinical workflows. Additionally, only one open-source LLM was utilized, and performance comparisons with other models were not conducted. In the future, benchmarking across multiple LLMs or optimizing prompt design tailored to the specific characteristics of pathology reports may further improve extraction accuracy. Furthermore, integrating probabilistic reasoning into the knowledge graph, such as converting microscopic findings into probability values, could enhance the processing of detailed findings from unstructured pathology reports within the knowledge graph framework. Despite these limitations, our approach demonstrates significant potential for supporting standardized AJCC staging and reducing medical errors through automated consistency validation. This methodology provides a foundation for developing comprehensive clinical decision support systems that can process unstructured medical text while maintaining patient privacy through local deployment strategies.

## 5. Conclusions

This study presents an integrated automated AJCC staging system for radical prostatectomy pathology reports by combining large language model-based information extraction with knowledge graph validation. The developed framework provides automated, privacy-preserving clinical decision support in cancer staging workflows and has potential for application to other oncologic domains.

## Figures and Tables

**Table 1:** Prioritized rule-based staging criteria for prostate cancer, according to the 8th edition of the AJCC [23].

Condition	AJCC Stage
M1 (any T, any N, any GG, any PSA)	IVB
N1 (any T, M0, any GG, any PSA)	IVA
Grade Group 5 (any T, N0, M0, any PSA)	IIIC
T3/T4 (N0, M0, GG 1–4, any PSA)	IIIB
PSA ≥ 20 (T1–T2, N0, M0, GG 1–4)	IIIA
T1–T2a, N0, M0, GG1, PSA < 10	I
T1–T2, N0, M0, GG1, PSA ≥10, <20	IIA
T1–T2, N0, M0, GG2, PSA < 20	IIB
T1–T2, N0, M0, GG3–4, PSA < 20	IIC

**Table 2:** Characteristics of the internal and external validation datasets.

Parameter	Datasets, *n* (%)		*p* Value
	Internal (*n* = 152)	External (*n* = 88)	
**WHO Grade Group**			<0.001
GG1	2 (1.3)	6 (6.8)	
GG2	70 (46.1)	30 (34.1)	
GG3	48 (31.6)	22 (25.0)	
GG4	4 (2.6)	13 (14.8)	
GG5	20 (13.2)	17 (19.3)	
-	3 (2.0)	0 (0.0)	
Not mentioned	5 (3.3)	0 (0.0)	
**T-Stage**			0.014
pT2a	2 (1.3)	0 (0.0)	
pT2b	3 (2.0)	1 (1.1)	
pT2c	72 (47.4)	29 (33.0)	
pT3a	47 (30.9)	34 (38.6)	
pT3b	22 (14.5)	21 (23.9)	
pT4	0 (0.0)	3 (3.4)	
-	6 (3.9)	0 (0.0)	
Not mentioned	0 (0.0)	0 (0.0)	
**N-Stage**			0.090
pN0	112 (73.7)	72 (81.8)	
pN1	14 (9.2)	11 (12.5)	
pNx	22 (14.5)	5 (5.7)	
Not mentioned	4 (2.6)	0 (0.0)	
**Serum PSA (95% CI)**	11.9 (8.0–17.2)	7.11 (5.0–10.085)	<0.001
**M-Stage**			NA
cM0	3 (2.0)	1 (1.1)	
pM0	0 (0.0)	15 (17.0)	
pMx	0 (0.0)	11 (12.5)	
Not mentioned	149 (98.0)	61 (69.3)	
**EPE**			0.002
Absent	83 (54.6)	30 (34.1)	
Present	67 (44.1)	58 (65.9)	
Not mentioned	2 (1.3)	0 (0.0)	
**SVI**			0.055
Absent	129 (84.9)	65 (73.9)	
Present	23 (15.1)	23 (26.1)	
**Perineural Invasion**			0.948
Absent	12 (7.9)	8 (9.1)	
Present	139 (91.4)	80 (90.9)	
Not mentioned	1 (0.7)	0 (0.0)	

**Table 3:** AJCC stage distribution and PSA value comparison in original versus imputed data.

AJCC Stage	Datasets, *n* (%)		*p* Value
	Internal *n* (%, 95% CI)	External *n* (%, 95% CI)	
IIA	0	3 (4.2, 1.4–11.5)	0.050
IIB	35 (28.7, 21.4–37.3)	15 (20.8, 13.1–31.6)	0.227
IIC	14 (11.5, 7.0–18.3)	6 (8.3, 3.9–17.0)	0.487
IIIA	7 (5.7, 2.8–11.4)	0	0.048
IIIB	38 (31.1, 23.6–39.8)	31 (43.1, 32.3–54.6)	0.094
IIIC	15 (12.3, 7.6–19.3)	8 (11.1, 5.7–20.4)	0.805
IVA	13 (10.7, 6.3–17.4)	9 (12.5, 6.7–22.1)	0.696
Overall	122 (100)	72 (100)	0.048

**Table 4:** LLM information extraction performance by parameters in the internal dataset (*n* = 150 cases).

Parameter	Correct	Accuracy	Precision	Recall	F1-Score
EPE	148	0.987	1.000	0.987	0.993
Histologic Subtype	139	0.927	1.000	0.927	0.962
Lymph Nodes with Metastasis	149	0.993	0.993	1.000	0.997
M-Stage	150	1.000	1.000	1.000	1.000
N-Stage	149	0.993	0.993	1.000	0.997
Number of Lymph Nodes examined	148	0.987	0.993	0.993	0.993
Percentage of Secondary Gleason Pattern	136	0.907	0.971	0.932	0.951
Perineural Invasion	147	0.980	1.000	0.980	0.990
Primary Gleason Pattern	148	0.987	1.000	0.987	0.993
Resection Margins	148	0.987	1.000	0.987	0.993
Secondary Gleason Pattern	148	0.987	1.000	0.987	0.993
SVI	150	1.000	1.000	1.000	1.000
PSA	140	0.933	0.933	1.000	0.966
T-Stage	144	0.960	0.980	0.980	0.980
Tertiary Gleason Pattern	149	0.993	1.000	0.993	0.997
WHO Grade Group	141	0.940	1.000	0.940	0.969
Overall (micro)	2334	0.973	0.992	0.981	0.986

**Table 5:** LLM information extraction performance by parameters in the external validation dataset (*n* = 88 cases).

Parameter	Correct	Accuracy	Precision	Recall	F1-Score
EPE	87	0.989	0.989	1.000	0.994
Histologic Subtype	78	0.886	0.897	0.987	0.940
Lymph Nodes with Metastasis	88	1.000	1.000	1.000	1.000
M-Stage	61	0.693	0.984	0.701	0.819
N-Stage	88	1.000	1.000	1.000	1.000
Number of Lymph Nodes examined	88	1.000	1.000	1.000	1.000
Percentage of Secondary Gleason Pattern	59	0.670	0.967	0.686	0.803
Perineural Invasion	88	1.000	1.000	1.000	1.000
Primary Gleason Pattern	87	0.989	1.000	0.989	0.994
Resection Margins	87	0.989	1.000	0.989	0.994
Secondary Gleason Pattern	87	0.989	1.000	0.989	0.994
SVI	88	1.000	1.000	1.000	1.000
PSA	87	0.989	0.989	1.000	0.994
T-Stage	88	1.000	1.000	1.000	1.000
Tertiary Gleason Pattern	75	0.852	1.000	0.852	0.920
WHO Grade Group	84	0.955	1.000	0.955	0.977
Overall (micro)	1320	0.938	0.990	0.947	0.968

**Table 6:** AJCC staging classification performance by stage using knowledge graph-based rule application in the internal dataset.

AJCC Stage	TP	FP	FN	Precision	Recall	F1-Score
IIB	33	1	2	0.971	0.943	0.957
IIC	13	2	1	0.867	0.929	0.897
IIIA	7	1	0	0.875	1.000	0.933
IIIB	38	4	0	0.905	1.000	0.950
IIIC	14	0	0	1.000	1.000	1.000
IVA	12	1	0	0.923	1.000	0.960
Unknown	22	2	8	0.917	0.733	0.815
Overall (macro)	-	-	-	0.922	0.944	0.930

**Table 7:** AJCC staging classification performance by stage using knowledge graph-based rule application in the external validation dataset.

AJCC Stage	TP	FP	FN	Precision	Recall	F1-Score
IIA	3	1	0	0.750	1.000	0.857
IIB	13	2	2	0.867	0.867	0.867
IIC	6	1	0	0.857	1.000	0.923
IIIB	31	4	0	0.886	1.000	0.939
IIIC	8	2	0	0.800	1.000	0.889
IVA	9	2	0	0.818	1.000	0.900
Unknown	5	1	11	0.833	0.313	0.455
Overall (macro)	-	-	-	0.830	0.883	0.833

**Table 8:** Types and frequency of staging and grade inconsistencies detected by knowledge graph validation in the internal dataset.

Error_Type	*n* (%)
T3a_stage_but_no_EPE	2 (40)
Gleason_7_inconsistent_with_GG3	1 (20)
SVI_present_should_be_T3b_or_higher	1 (20)
Gleason_7_inconsistent_with_GG2	1 (20)

## Data Availability

The data presented in this study are available in Zenodo at https://doi.org/10.5281/zenodo.14175293, reference number [18], and Mendeley Data at https://data.mendeley.com/datasets/hyg5xkznpx/1, reference number [20]. These data were derived from the following resources available in the public domain: The Cancer Genome Atlas (TCGA, https://www.cancer.gov/ccg/research/genome-sequencing/tcga) for reference number [20].

## Abbreviations

AJCC	American Joint Committee on Cancer
EPE	Extraprostatic extension
FN	False Negative
FP	False Positive
GS	Gleason scores
ICCR	International Collaboration on Cancer Reporting
ISUP	International Society of Urological Pathology
LLM	Large language model
PSA	Prostate-specific antigen
RP	Radical prostatectomy
SVI	Seminal vesicle invasion
TCGA	The Cancer Genome Atlas
TP	True Positive
TNM	Tumor-Node-Metastasis
WHO	World Health Organization

## Appendix A

### Appendix A.1

Prompt for automated pathological parameter extraction using GPT-4.1

** **

You are a senior genitourinary pathologist. Extract ONLY the five parameters listed below from the user supplied pathology report and return a **STRICTLY valid single line JSON object** (no markdown, no commentary).

** **

──────── OUTPUT FORMAT ────────

{

  “extracted” : {

  “Serum_PSA_ng_per_mL” : <float> | “Not mentioned”,

  “M_Stage” : “cM0” | “cM1” | “pM0” | “pM1” | “Not mentioned”,

  “Extraprostatic_Extension_EPE” : “Present” | “Absent” | “Not mentioned”,

  “Seminal_Vesicle_Invasion_SVI” : “Present” | “Absent” | “Not mentioned”,

  “Perineural_Invasion_Pn” : “Present” | “Absent” | “Not mentioned”

  },

  “evidence” : {

  “Serum_PSA_ng_per_mL” : “<quote or null>“,

  “M_Stage” : “<quote or null>“,

  “Extraprostatic_Extension_EPE” : “<quote or null>“,

  “Seminal_Vesicle_Invasion_SVI” : “<quote or null>“,

  “Perineural_Invasion_Pn” : “<quote or null>“

  },

  “confidence” : {

  “Serum_PSA_ng_per_mL” : 0.00 1.00,

  “M_Stage” : 0.00 1.00,

  “Extraprostatic_Extension_EPE” : 0.00 1.00,

  “Seminal_Vesicle_Invasion_SVI” : 0.00 1.00,

  “Perineural_Invasion_Pn” : 0.00 1.00

  }

}

──────── EXTRACTION RULES ────────

1 Use **only** the allowed categories/number formats above.
2 PSA : choose the **pretreatment serum PSA** value; strip “ng/mL”; output as float.
3 If the report lacks clear info → set value = “Not mentioned”, evidence = null, confidence = 0.0.
4 Quote evidence verbatim; maximum 25 words.
5 Return JSON only—no other text.

### Appendix A.2

Prompt for Parameter Extraction for External Validation Dataset Annotation Using GPT-4.1

You are a senior genitourinary pathologist.

Extract ONLY the parameters listed below from the user-supplied pathology report

and return a **STRICTLY valid single-line JSON object** (no markdown, no commentary).

** **

──────── OUTPUT FORMAT ────────

{

  “extracted”: {

  “WHO_ISUP_Grade_Group”: “GG1” | “GG2” | “GG3” | “GG4” | “GG5” | “Not mentioned”,

  “T_Stage_TNM”: “pT0” | “pT2a” | “pT2b” | “pT2c” | “pT3a” | “pT3b” | “pT4” | “Not mentioned”,

  “N_Stage_TNM”: “pNx” | “pN0” | “pN1” | “Not mentioned”,

  “Number_of_Lymph_Nodes_examined”: <integer> | “Not mentioned”,

  “Lymph_Nodes_with_Metastasis”: <integer> | “Not mentioned”,

  “Resection_Margins”: “R0” | “R1” | “Rx” | “Not mentioned”,

  “Histologic_Subtype”: “acinar” | “ductal” | “mixed” | “Not mentioned”,

  “Primary_Gleason_Pattern”: 3 | 4 | 5 | “Not mentioned”,

  “Secondary_Gleason_Pattern”: 3 | 4 | 5 | “Not mentioned”,

  “Tertiary_Gleason_Pattern”: 3 | 4 | 5 | “Not mentioned”,

  “Percentage_of_Secondary_Gleason_Pattern”: <integer> | “Not mentioned”,

  “Serum_PSA_ng_per_mL”: <float> | “Not mentioned”,

  “M_Stage_TNM”: “cM0” | “cM1” | “pM0” | “pM1” | “Not mentioned”,

  “Extraprostatic_Extension_EPE”: “Present” | “Absent” | “Not mentioned”,

  “Seminal_Vesicle_Invasion_SVI”: “Present” | “Absent” | “Not mentioned”,

  “Perineural_Invasion_Pn”: “Present” | “Absent” | “Not mentioned”

  },

  “evidence”: {

  “WHO_ISUP_Grade_Group”: “<quote or null>“,

  “T_Stage_TNM”: “<quote or null>“,

  “N_Stage_TNM”: “<quote or null>“,

  “Number_of_Lymph_Nodes_examined”: “<quote or null>“,

  “Lymph_Nodes_with_Metastasis”: “<quote or null>“,

  “Resection_Margins”: “<quote or null>“,

  “Histologic_Subtype”: “<quote or null>“,

  “Primary_Gleason_Pattern”: “<quote or null>“,

  “Secondary_Gleason_Pattern”: “<quote or null>“,

  “Tertiary_Gleason_Pattern”: “<quote or null>“,

  “Percentage_of_Secondary_Gleason_Pattern”: “<quote or null>“,

  “Serum_PSA_ng_per_mL”: “<quote or null>“,

  “M_Stage_TNM”: “<quote or null>“,

  “Extraprostatic_Extension_EPE”: “<quote or null>“,

  “Seminal_Vesicle_Invasion_SVI”: “<quote or null>“,

  “Perineural_Invasion_Pn”: “<quote or null>“

  },

  “confidence”: {

  “WHO_ISUP_Grade_Group”: 0.00–1.00,

  “T_Stage_TNM”: 0.00–1.00,

  “N_Stage_TNM”: 0.00–1.00,

  “Number_of_Lymph_Nodes_examined”: 0.00–1.00,

  “Lymph_Nodes_with_Metastasis”: 0.00–1.00,

  “Resection_Margins”: 0.00–1.00,

  “Histologic_Subtype”: 0.00–1.00,

  “Primary_Gleason_Pattern”: 0.00–1.00,

  “Secondary_Gleason_Pattern”: 0.00–1.00,

  “Tertiary_Gleason_Pattern”: 0.00–1.00,

  “Percentage_of_Secondary_Gleason_Pattern”: 0.00–1.00,

  “Serum_PSA_ng_per_mL”: 0.00–1.00,

  “M_Stage_TNM”: 0.00–1.00,

  “Extraprostatic_Extension_EPE”: 0.00–1.00,

  “Seminal_Vesicle_Invasion_SVI”: 0.00–1.00,

  “Perineural_Invasion_Pn”: 0.00–1.00

  }

}

** **

──────── EXTRACTION RULES ────────

1. Use **only** the allowed categories/number formats above.
2. **WHO/ISUP Grade Group**: Look for “Grade Group”, “ISUP grade”, “GG1-5”, or equivalent grading terminology.
3. **T-Stage**: Focus on pathologic T-stage (pT). Look for tumor extent and capsular involvement.
4. **N-Stage**: Extract nodal involvement status (pNx, pN0, pN1).
5. **Lymph Nodes**:
  - Count total examined nodes separately from positive nodes
  - Extract exact numbers when available
6. **Resection Margins**:
  - R0 = negative/clear margins
  - R1 = positive margins
  - Rx = cannot be assessed
7. **Histologic Subtype**: Look for adenocarcinoma subtypes (acinar, ductal, mixed).
8. **Gleason Patterns**:
  - Extract primary (predominant), secondary, and tertiary patterns
  - Look for percentage of secondary pattern when mentioned
9. **PSA**: Use **pretreatment serum PSA** value; strip “ng/mL” units; output as float.
10. **M-Stage**: Look for metastasis status (clinical cM or pathologic pM).
11. **Extensions/Invasions**: Determine presence or absence of:
  - Extraprostatic extension (EPE)
  - Seminal vesicle invasion (SVI)
  - Perineural invasion (Pn)
12. **General Rules**:
  - If report lacks clear information → set value = “Not mentioned”, evidence = null, confidence = 0.0
  - Quote evidence verbatim; maximum 25 words per quote
  - Return JSON only—no other text or formatting
  - Confidence should reflect certainty of extraction (1.0 = completely certain, 0.0 = not found)

### Appendix A.3

Mistral-Small-3.2-24B-Instruct prompt for extracting 16 parameters from pathology reports

** **

You are a senior genitourinary pathologist with expertise in prostate cancer. Extract ONLY the 16 parameters listed below from the pathology report and return a STRICTLY valid JSON object.

CRITICAL INSTRUCTIONS:

1. Use EXACTLY the specified answer options for each parameter
2. If information is not clearly stated, use “Not mentioned”
3. For integer fields, provide only the numeric value
4. For float fields (PSA), provide only the numeric value
5. Return ONLY the JSON object - no explanations, no markdown, no commentary

OUTPUT FORMAT:

{

  “WHO_ISUP_Grade_Group”: “GG1” | “GG2” | “GG3” | “GG4” | “GG5” | “Not mentioned”,

  “T_Stage_TNM”: “pT0” | “pT2a” | “pT2b” | “pT2c” | “pT3a” | “pT3b” | “pT4” | “Not mentioned”,

  “N_Stage_TNM”: “pNx” | “pN0” | “pN1” | “Not mentioned”,

  “Number_of_Lymph_Nodes_examined”: <integer> | “Not mentioned”,

  “Lymph_Nodes_with_Metastasis”: <integer> | “Not mentioned”,

  “Resection_Margins”: “R0” | “R1” | “Rx” | “Not mentioned”,

  “Histologic_Subtype”: “acinar” | “ductal” | “mixed” | “Not mentioned”,

  “Primary_Gleason_Pattern”: 3 | 4 | 5 | “Not mentioned”,

  “Secondary_Gleason_Pattern”: 3 | 4 | 5 | “Not mentioned”,

  “Tertiary_Gleason_Pattern”: 3 | 4 | 5 | “Not mentioned”,

  “Percentage_of_Secondary_Gleason_Pattern”: <integer> | “Not mentioned”,

  “Serum_PSA_ng_per_mL”: <float> | “Not mentioned”,

  “M_Stage_TNM”: “cM0” | “cM1” | “pM0” | “pM1” | “Not mentioned”,

  “Extraprostatic_Extension_EPE”: “Present” | “Absent” | “Not mentioned”,

  “Seminal_Vesicle_Invasion_SVI”: “Present” | “Absent” | “Not mentioned”,

  “Perineural_Invasion_Pn”: “Present” | “Absent” | “Not mentioned”

}

** **

EXTRACTION GUIDELINES:

- WHO/ISUP Grade Group: Look for Grade Group, ISUP grade, or equivalent grading
- T-Stage: Focus on pathologic T-stage (pT), primary tumor extent
- N-Stage: Look for nodal involvement status
- Lymph nodes: Count examined nodes and positive nodes separately
- Margins: R0 = negative, R1 = positive, Rx = cannot be assessed
- Gleason: Extract primary, secondary, and tertiary patterns with percentages
- PSA: Use pretreatment serum PSA value, remove units

Extensions/Invasions: Determine presence or absence of specific invasions

** **

Analyze the following pathology report:

**Table A1:** Representative examples of GPT-4.1 extraction output with confidence scores and supporting evidence for data element expansion.

Parameter	Extracted Value	Confidence	Evidence Span
PSA level	14	1.00	PSA 14 ng/mL.
M-stage	cM0	0.95	Staging without metastases.
EPE	Present	1.00	Right-sided capsular perforation with infiltration of the adjacent fatty/connective tissue.
SVI	Absent	1.00	No infiltration of the seminal vesicles and the ductus deferens segments on both sides.
Perineural invasion	Present	1.00	Extensive perineural sheath invasion.

**Table A2:** Representative examples of GPT-4.1 output for annotating external validation datasets.

Parameter	Extracted Value	Confidence	Evidence Span
EPE	Present	1.00	EXTRAPROSTATIC EXTENSION: Yes-Established (>0.8 mm).
Histologic Subtype	acinar	0.95	TUMOR HISTOLOGY: Adenocarcinoma NOS.
Lymph Nodes with Metastasis	0	1.00	LYMPH NODES POSITIVE: o.
M-Stage	pMx	0.90	M STAGE, PATHOLOGIC: pMX.
N-Stage	pN0	1.00	N STAGE, PATHOLOGIC: pNO
Number of Lymph Nodes examined	11	1.00	LYMPH NODES EXAMINED: 11
Percentage of Secondary Gleason Pattern	10	1.00	Gleason pattern 5 constitutes approximately 10% of the tissues evaluated.
Perineural Invasion	Present	1.00	MULTIFOCAL PERINEURAL INVASION BY THE CARCINOMA IS SEEN.
Primary Gleason Pattern	4	1.00	PRIMARY GLEASON GRADE: 4.
Resection Margins	R0	1.00	All surgical margins free of tumor. RESIDUAL TUMOR: R0.
Secondary Gleason Pattern	5	1.00	SECONDARY GLEASON GRADE: 5.
SVI	Absent	1.00	BILATERAL SEMINAL VESICLES, NO EVIDENCE OF CARCINOMA SEEN.
PSA	35	1.00	PSA value: 35.
T-Stage	pT3a	1.00	T STAGE, PATHOLOGIC: pT3a
Tertiary Gleason Pattern	3	1.00	Gleason pattern 3 constitutes approximately 35%
WHO Grade Group	GG5	1.00	Gleason score 4 + 5 a 9
